# Rasburicase in treating tumor lysis syndrome: An umbrella review

**DOI:** 10.1016/j.cpt.2023.07.001

**Published:** 2023-07-20

**Authors:** Kamran Mahfooz, Haris Sohail, Ani Gvajaia, Uroosa Arif, Daisy Grewal, Monica Reddy Muppidi, Vanya Vohra, Aamir Tarique, Advait Vasavada

**Affiliations:** a Department of Internal Medicine, Lincoln Medical Center, Community Hospital, New York, 10451, USA; b Department of Internal Medicine, St. Georges University, St. Georges, Grenada; c Department of Pediatrics, St Barnabas Hospital, Bronx, NY, 10457, USA; d Department of Medicine, ESIC Medical College, Faridabad, 121001, India; e Department of Medicine, MP Shah Medical College, Jamnagar, 361008, India

**Keywords:** Tumor lysis, Hematologic urgency, Rasburicase, Drug therapy

## Abstract

Tumor lysis syndrome (TLS) remains a debilitating cause of hospitalization and death in patients with cancer and is a significant challenge for healthcare providers despite advancements in its management. This umbrella review analyzed the results of meta-analyses on the use of rasburicase in the treatment of patients with cancer. A literature search was performed of five databases (PubMed, Google Scholar, Cochrane Library, Scopus, Global Index Medicus, and ScienceDirect) for articles with full texts available online. A measurement tool to assess systematic reviews 2 (AMSTAR 2) was used to assess the quality of the included studies, and Review Manager software was used to conduct all statistical analyses. The systematic search identified eight relevant meta-analyses, with primary analyses including outcome data that analyzed mortality, renal failure, and comparisons with allopurinol. The pooled data showed that rasburicase effectively reduced TLS development and serum uric acid levels in children and adults with malignancies. Most outcomes did not differ significantly compared with those of allopurinol. Future trials should focus on the cost-effectiveness of rasburicase compared to that of allopurinol while including high-, intermediate-, and low-risk patients. Rasburicase is safe and effective for managing patients with TLS. However, recent large-scale meta-analyses have reported conflicting results. Most meta-analyses were graded as low to critically low as per AMSTAR 2. The analysis revealed that the benefit of rasburicase did not differ significantly from that of allopurinol, which has higher cost-effectiveness and fewer side effects.

## Highlights

• Rasburicase is safe and effective in managing tumor lysis syndrome.
• Recent meta-analyses reported conflicting results for rasburicase *vs.* allopurinol.
• Cost-effectiveness and side effects are potential concerns in rasburicase use.

## Graphical abstract

**Figure:**
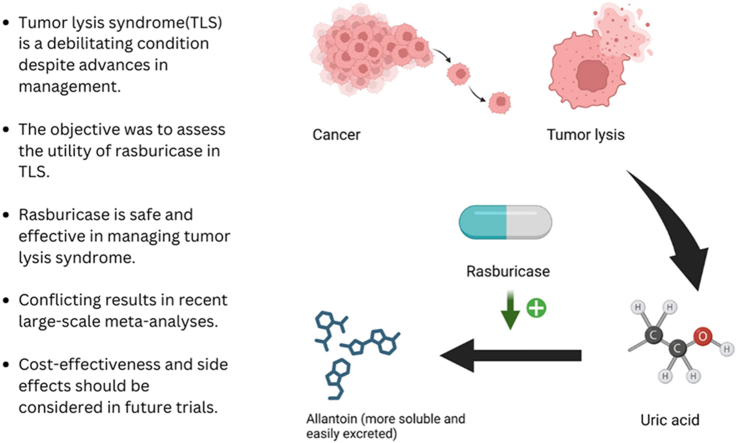

## Introduction

Tumor lysis syndrome (TLS) is the most common hematologic cancer emergency and is equally prevalent in children and adults.^1^^,^^2^ TLS was initially identified in patients with acute leukemia and non-Hodgkin’s lymphoma; however, since then, it has become more prevalent and is associated with several other types of cancer.^3^^,^^4^ TLS occurs when tumor cells burst, leading to the release of large amounts of phosphate, potassium, and uric acid into the circulation.^5^

Several factors can trigger TLS, including acute lymphoblastic leukemia and high-grade lymphomas. TLS can occur in patients receiving cytotoxic therapy during the course of the disease.^6^ Other factors include a high proliferative rate and sensitivity to cytotoxic therapy, which make the tumor more likely to burst, leading to TLS.^6^ Signs of at-risk patients are bulky malignancies; increased serum phosphorus, uric acid, and potassium levels; proliferating tumors; and large tumor surface area after therapy.^7^^,^^8^ Additionally, the latest treatment options for chronic lymphocytic leukemia, such as oral kinase inhibitors and venetoclax, B-cell lymphoma-2 protein inhibitors, ibrutinib, and idelalisib, can also lead to TLS because of the extreme sensitivity of such treatment and enhanced tumor growth.^9^

The diagnosis of TLS requires two laboratory tests and one clinical criterion. Laboratory TLS (LTLS) is often characterized by one of three conditions. In the first condition, uric acid levels are normal, and potassium and phosphate levels are 25% above the baseline level. The second condition is characterized by normal uric acid levels and potassium and phosphate levels that exceed the upper limit of the normal levels. In the third condition, uric acid levels are increased by 25% from baseline or exceed the upper normal limit, along with increased phosphate or potassium levels. Combined with these laboratory findings, several clinical criteria are often present due to the deposition of toxic metabolites in the blood and the underlying electrolyte imbalance. One of the clinical criteria is an increased creatinine level ≥1.5 times the upper normal limit, usually manifesting as anuria or oliguria. Cardiac arrhythmia or sudden death resulting from hyperkalemia is also common.^10^^,^^11^ The signs of TLS can manifest as early as a few hours after the chemotherapy initiation but more commonly occur 24–48 h later.^11^, ^12^, ^13^, ^14^, ^15^, ^16^, ^17^, ^18^, ^19^, ^20^, ^21^

The prophylactic management of TLS in high-risk patients includes treatments to reduce uric acid production (using allopurinol), uric acid removal through enzymatic action (using rasburicase), and vigorous hydration.^11^ Additionally, external administration of phosphorus and potassium is avoided. Close monitoring is required during prophylactic management to detect and treat any metabolic abnormalities before they start causing symptoms.

The current treatment options for TLS and uric acid management are allopurinol and rasburicase. Allopurinol inhibits the enzymatic activity of xanthine oxidase, thereby preventing uric acid formation and reducing urinary and serum uric acid levels.^22^ Rasburicase, obtained from modified *Aspergillus flavus* and is usually expressed in *Saccharomyces cerevisiae*,^23^ is a urate oxidase that converts uric acid to a non-pathological and 10 times more soluble allantoin, thus reducing serum uric acid levels.^22^ The conversion of uric acid to allantoin via the enzymatic action of rasburicase leads to the production of carbon dioxide and hydrogen peroxide.^24^

Allopurinol decreases uric acid production during TLS but has no significant effect on the current uric acid levels. In contrast, rasburicase not only reduces the current uric acid levels but also decreases the risk of tumor lysis.^22^ Therefore, rasburicase is used for the initial management of TLS and uric acid levels in high-risk patients (children and adults) with solid tumors, leukemia, and lymphoma.^23^

The overall half-life of rasburicase is 18 h, depending on the dosing regimen. For instance, the half-life of a 0.15 mg/kg dose regimen is 16 h, while that of a 0.2 mg/kg dose regimen is 21 h. Observation after 1 d of treatment indicated no rasburicase deposition.^23^^,^^25^ Rasburicase is degraded in the body through peptide hydrolysis; thus, its clearance from the body is independent of hepatic and renal functions (usually impaired during TLS).^26^

The recommended dose of rasburicase is 0.15 mg/kg/day or 0.2 mg/kg/day for children and 0.2 mg/kg/day for adults. The course should be 5 days, and medication beyond 5 days is usually not recommended. The first dose of rasburicase should be administered 4–24 h before initiating chemotherapy/radiotherapy, and it is administered intravenously for 30 min.^23^ In contrast to weight-based rasburicase dosage, a single-dose regimen is recommended. The literature has established that a single dose of 7.5 mg/day is the most efficacious in minimizing the risk of laboratory and clinical TLS.^27^

Extensive studies established the efficacy of rasburicase for TLS development and control of uric acid levels during the disease course.^13^, ^14^, ^15^, ^16^, ^17^, ^18^, ^19^, ^20^, ^21^ However, the area lacks a review that evaluates information and the quality of overarching evidence provided by the systematic reviews on this topic. Hence, this umbrella review aimed to synthesize the results of systematic reviews exploring the efficacy of rasburicase in TLS development. The primary outcomes analyzed were TLS development and uric acid levels, while the secondary outcomes were adverse events, AKI, creatinine levels, mortality, and economic outcomes.

## Methods

### Search strategy

Electronic databases (PubMed, Google Scholar, Cochrane Library, Scopus, Global Index Medicus, and ScienceDirect) were searched from their earliest dates until February 2023. Database-specific subject headings were also used, which were mapped onto the search terms (listed below). These online literature searches were supplemented by checking for any additional potentially eligible papers cited by the included articles and the reference lists of the relevant reviews. The keywords and combinations used in the search were as follows:

PubMed: (“tumour lysis syndrome”[All Fields] OR “tumor lysis syndrome”[MeSH Terms] OR (“tumor”[All Fields] AND “lysis”[All Fields] AND “syndrome”[All Fields]) OR “tumor lysis syndrome”[All Fields]) AND (“rasburicase”[Supplementary Concept] OR “rasburicase”[All Fields] OR (“urate oxidase”[MeSH Terms] OR (“urate”[All Fields] AND “oxidase”[All Fields]) OR “urate oxidase”[All Fields])) AND (“systematic review”[Publication Type] OR “systematic reviews as topic”[MeSH Terms] OR “systematic review”[All Fields] OR (“review”[Publication Type] OR “review literature as topic”[MeSH Terms] OR “review”[All Fields]) OR (“meta analysis”[Publication Type] OR “meta analysis as topic”[MeSH Terms] OR “meta analysis”[All Fields])).

Google Scholar: (‘tumor lysis syndrome’) AND (‘Rasburicase’ OR ‘urate oxidase’) AND (‘systematic review’ OR ‘review’ OR ‘meta-analysis’).

The advanced search function was used in Google Scholar as follows: Search for articles containing all the words: Rasburicase Tumor Lysis Syndrome Return the articles authored by [left]. Published in Journal: [Left blank]. Where my words occur: in the title of the article.

Cochrane Library: (Title/Abstract/Keywords): “Rasburicase”, (Title/Abstract/Keywords): “Tumor Lysis Syndrome”, “Rasburicase” AND “Tumor Lysis Syndrome”.

Scopus: (TITLE-ABS-KEY): “Rasburicase”, (TITLE-ABS-KEY): “Tumor Lysis Syndrome”, “Rasburicase” AND “Tumor Lysis syndrome”. The filters used were the date range, language, and document type.

Global Index Medicus: (Title/Abstract/Keywords): “Rasburicase”, (Title/Abstract/Keywords): “Tumor Lysis Syndrome”, “Rasburicase” AND “Tumor Lysis Syndrome”; ScienceDirect: (Title/Abstract/Keywords): “Rasburicase”, (Title/Abstract/Keywords): “Tumor Lysis Syndrome”, “Rasburicase” AND “Tumor Lysis Syndrome”.

Data were screened in two steps. First, two independent reviewers (K.M. and A.G.) screened titles and abstracts based on the inclusion and exclusion criteria. Articles were retained in cases of uncertainty. Second, the full-text articles were screened. The titles/abstracts and full texts were parallelly screened at both stages. Discrepancies were resolved through discussions and intervention by a third person (H.S.).

### Inclusion and exclusion criteria

Both published and gray literature were included. Studies were included if they met the population, intervention, comparator, or outcome (PICO) criteria as follows: (1) population: adults and children with malignancies; (2) intervention: rasburicase; (3) comparator: allopurinol or any other control group; and (4) outcomes: TLS development and serum uric acid level (secondary outcomes: AKI, mortality, adverse events, renal replacement therapy [RRT], and economic outcomes). Studies were excluded if they were individual studies, case reports, conference proceedings, editorials, letters, commentaries, non-English articles, or if the full text was unavailable.

### Risk of bias

A risk of bias assessment of the included reviews was conducted using a measurement tool to assess systematic reviews 2 (AMSTAR 2).^28^^,^^29^ AMSTAR 2 is a critical appraisal tool for systematic reviews and meta-analyses that includes randomized controlled trials (RCTs), non-randomized studies, or both to assess healthcare interventions. The tool consists of 16 review questions, review methodology (inclusion/exclusion criteria, search strategy, and article screening), risk of bias assessment, quantitative synthesis in the review, and ethical concerns. The questions were answered as yes, no, or partial. “Yes” responses were scored as 1, and the responses were categorized based on the method described by the Canadian Agency for Drugs and Technologies in Health (CADTH).^28^ AMSTAR 2 uses a critical appraisal approach with four overall confidence categories: high, moderate, low, and critically low.^29^

### Data extraction

Quantitative information was extracted independently by at least two reviewers (D.G. and V.V.) using a data extraction spreadsheet. Data related to the database and period of search for the review, sample characteristics of the included studies, study characteristics, interventions, outcomes, and results were extracted. Any disagreements were resolved after discussion among team members. The authors were contacted to provide additional information when data were missing or unclear. The primary outcomes were TLS development and serum uric acid levels.

### Data analysis

The inverse variance method was used to pool the data. A meta-analysis was performed using Review Manager v5.4 (RevMan) software. Effect sizes are expressed as the log of the odds ratio (OR) with a 95% confidence interval (CI). This method assigned more weight to studies with smaller standard errors (or greater precision) and less weight to those with larger standard errors (or less precision). In the context of our review, studies with larger sample sizes and lower variability in their outcomes were considered more precise; hence, they contributed more to the pooled estimates. By taking the logarithm of the OR, the data were transformed to a scale more amenable to statistical analysis. This calculation rendered the data more symmetrically distributed and suitable for meta-analyses. We then back-transformed the pooled estimate to obtain the overall OR by calculating the exponential (anti-log) of the pooled log OR. In this inverse variance methodology, the overall estimate was influenced more by studies with smaller variances (greater precision) than by those with larger variances (lower precision).

## Results

**Figure 1:**
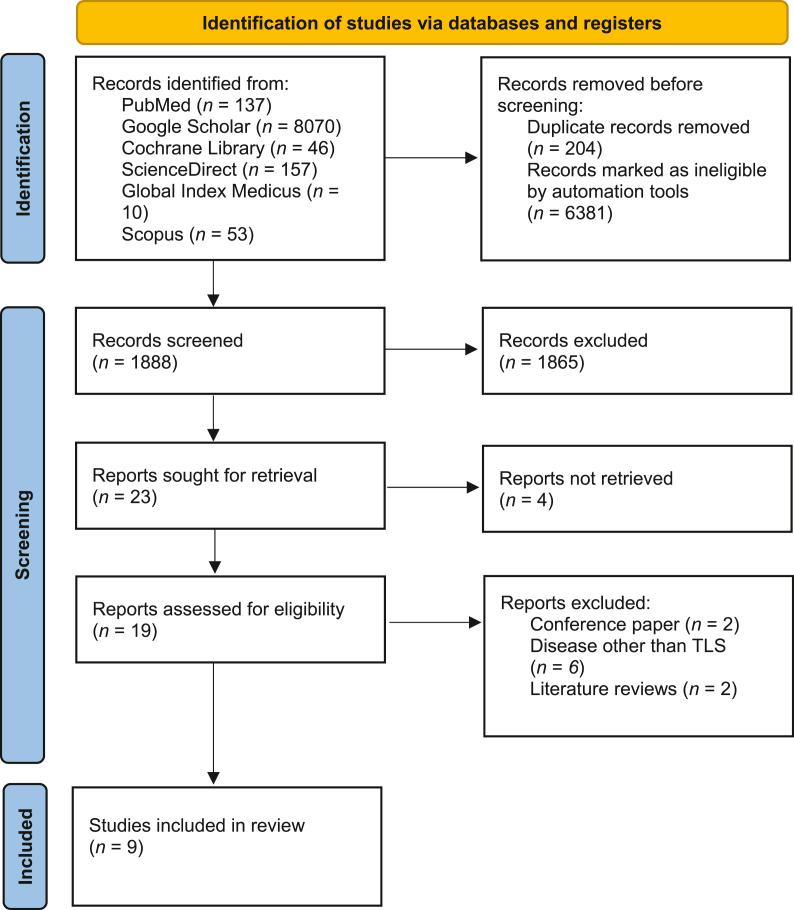
PRISMA flow chart of the study.^30^ PRISMA: Preferred reporting items for systematic reviews and meta-analyses; TLS: Tumor lysis syndrome.

The process of searching databases and screening results is illustrated in the preferred reporting items for systematic reviews and meta-analyses (PRISMA) flowchart. Nine studies were included in the umbrella review [Figure 1]. Table 1 depicts the study characteristics of the included meta-analyses.

**Figure 2:**
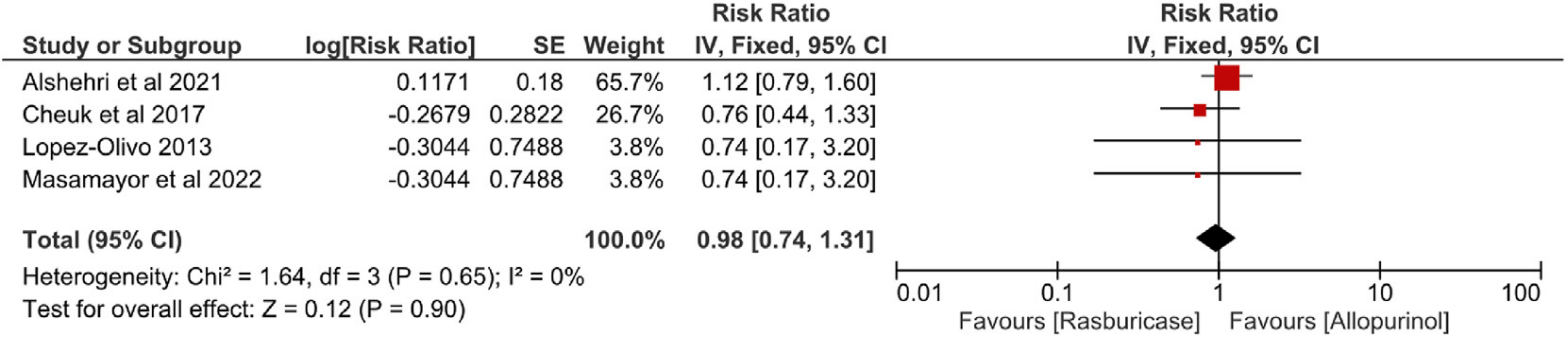
Efficacy of rasburicase *vs.* allopurinol to prevent the development of TLS. CI: Confidence interval; df: Degrees of freedom; IV: Inverse variance; SE: Standard error; TLS: Tumor lysis syndrome.

**Figure 3:**
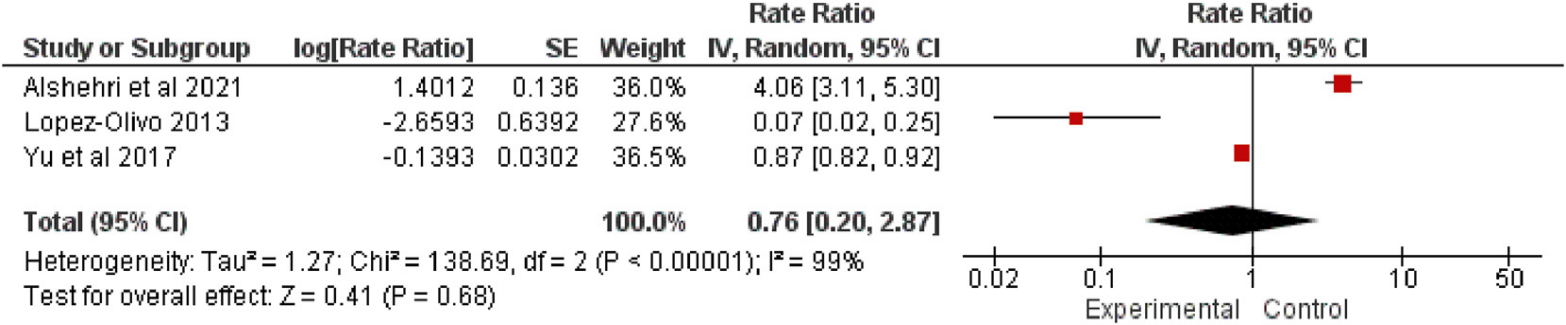
Efficacy of rasburicase against TLS development. CI: Confidence interval; df: Degrees of freedom; IV: Inverse variance; SE: Standard error; TLS: Tumor lysis syndrome.

**Table 1:** Study characteristics of the included systematic reviews.

Author (year)	Databases and period	Sample characteristics	Study characteristics (number of studies)	Intervention	Outcome (number of studies)	Results	AMSTAR 2 score
Lopez-Olivo et al., 2013^31^	MEDLINE, EMBASE, Cochrane Library, and Web of Science Inception till August 7, 2012	1261 adult patients	K = 21 (24 publications) Randomized controlled trials (4) Observational Studies (17) RoB: Cochrane Risk of Bias Assessment tool	Intervention: Rasburicase for TLS Control: Allopurinol	TLS development (3) Percentage of patients showing improvement (1) Total adverse events (11) Acute kidney failure (14) Deaths (9) Serum uric acid (20) Creatinine levels (6)	No difference in TLS development between the experimental and control groups in controlled trials. In observational studies, 7.40% of patients had TLS even after rasburicase treatment (95% CI = 1.70–16.70%), and 93.4% of patients achieved normal serum and uric acid levels after treatment. TLS development: RR (95% CI) = 0.07 (0.02–0.17) Uric acid level: RR (95% CI) = 0.93 (0.92–0.95) Adverse events: RR (95% CI) = 0.03 (0.02–0.04) AKI: RR (95% CI) = 0.04 (0.03–0.06) Death: RR (95% CI) = 0.03 (0.01–0.05)	9
Yu et al., 2017^32^	PubMed, MEDLINE, Web of Science, the Cochrane Library, and the ClinicalTrials.gov Inception till July 6, 2016	Adults = 906 Children = 92	K = 19 Adult studies: Retrospective (14) RCTs (1) Child studies: Observational (4) RoB: Cochrane Risk of Bias Assessment tool Countries: USA (18) Singapore (1)	Single doses of 1.5, 3, 4.5, 6, and 7.5 mg of rasburicase Comparison: weight-based single doses of 0.05 and 0.15 mg/kg of rasburicase	Adult studies: Response rate at 24 h (15) Mean reduction in serum uric acid level (10) Creatinine at 24 h (Insufficient data) Total adverse events (15) Incidence of the requirement for RRT (12) Mortality rate (insufficient data) Child studies: Mean reduction in serum uric acid level (4)	Response rates: 6 mg: 90.00% (95% CI = 0.82–0.97) 7.5 mg: 98.60% (95% CI = 0.90–1.01) 0.15 mg/kg: 93.60% (95% CI = 0.84–1.07) 6 mg and 0.15 mg/kg were most effective in reducing uric acid levels, with mean reductions of 8.45 mg/dL (95% CI = 7.51–9.38) and 10 mg/dL (95% CI = 8.58–11.42), respectively TLS: ER (95% CI) = 0.87 (0.82–0.93) Uric acid level: ER (95% CI) = 7.0 (5.84–8.17) Renal replacement treatment: ER (95% CI) = 0.65 (0.03–0.10)	9
Dinnel et al., 2015^33^	MEDLINE Inception till August 2014	Not provided	K = 98 Retrospective (26) Prospective (22) Reviews (50) RoB: Not conducted	Rasburicase for TLS	Patient-centered outcomes: PUA reduction (4)Prevention of LTLS/CTLS (8) Reduction of AKI incidence (8) Need for renal replacement therapy (RRT) (8) Intensive care unit (ICU) admission or length of stay (2) Mortality (48) Economic outcomes: Dose reduction (12)	Rasburicase results in the reduction of PUA levels and is superior to allopurinol in terms of PUA reduction. Rasburicase was also superior to allopurinol in reducing the risk for LTLS, CTLS, and RRT. Results also indicate that rasburicase is more cost-effective as it reduces the duration of ICU stay; however, the evidence of its cost-effectiveness is not robust. Moreover, there was no robust evidence to suggest that rasburicase reduces mortality in adults. Concerning the economic outcomes, studies suggest that single-dose rasburicase (SDR) is effective for TLS.	4
Feng et al., 2013^34^	PubMed, ScienceDirect, and ClinicalTrials.gov Inception till May 2012	Age range: 33–66 years Patients: 269	K = 10 Retrospective studies (8) Prospective (2) RoB: Not conducted	Single dose: 3 mg–7.5 mg Weight-based dosage: 0.05 mg/kg to 0.2 mg/kg Comparison group: Allopurinol at 300 mg for 5 days	Response rate: Serum uric acid level	A single dose of rasburicase was significantly better than was allopurinol (OR = 3.83, 95% CI = 2.17–6.76); however, the response rate did not differ between single and daily doses of rasburicase. Concerning the uric acid level, allopurinol failed to control the uric acid level, and the daily dose resulted in a greater reduction of the uric acid level than that with a single dose of rasburicase. Concerning economic outcomes, single-dose rasburicase was the most cost-efficient.	3
Masamayor et al., 2022^35^	PubMed, the Cochrane Library, and Clinicaltrials.gov Inception till December 2021	Participants: 633	K = 3 RCT (3) RoB: Cochrane Risk of Bias Assessment tool	Allopurinol, febuxostat, or rasburicase alone or in combination Comparison: intravenous hydration with crystalloid solutions and sodium bicarbonate	Incidence of laboratory and clinical TLS; Adverse events Serum uric acid levels	Rasburicase was more effective in preventing laboratory TLS than was allopurinol (RR = 0.74 (95% CI = 0.17–3.22). Rasburicase was the most effective agent in preventing laboratory TLS and maintaining low serum uric acid levels compared to the effects of allopurinol (MD = −569, 95% CI = −796.38 to −341.62) and febuxostat (MD = −426.08, 95% CI = −702.64 to −149.51). The most commonly reported adverse events were related to the blood, lymphatic, and gastrointestinal systems.	10
Bose et al., 2011^36^	MEDLINE Inception till February 2011	Not provided	K = 28 Retrospective (10) RCTs (5) Observation (6) Prospective (4) Case series (3) RoB: Not conducted	A single dose of rasburicase	Response rate (6) Uric acid level (23) Tolerability (2) Creatinine (1) Renal outcome (2)	Rasburicase significantly reduced serum uric acid levels even after the first dose. A high response rate to rasburicase was observed among patients at high risk of TLS	2
Alakel et al., 2016^37^	MEDLINE	Not provided	K = 10 Prospective (2) Retrospective (8) RoB: Not conducted	A single dose of rasburicase	Not mentioned	A single dose of rasburicase was effective for patients at high risk of TLS. No difference between weight-based and fixed single doses of rasburicase	1
Cheuk et al., 2017^38^	Cochrane Central Register of Controlled Trials (CENTRAL), MEDLINE/PubMed (1966 to March 14, 2016), Embase (Ovid) (1980 to March 14, 2016), and CINAHL (EBSCO) (1982 to March 14, 2016)	Intervention: *N*: 27 + 30+12 + 16 = 85 Control: *N*: 25 + 30+14 + 16 = 85	K = 4 RCT (2) CCT (2) RoB: Cochrane Risk of Bias Assessment Tool RoB v2	Intervention: Rasburicase Comparison: Allopurinol	All-cause mortality (4) Mortality due to TLS (4) Incidence of renal failure requiring RRT (4) Uric acid level (4) Adverse events (4)	No difference in mortality, mortality due to TLS, renal failure, and adverse events. Significant difference in the reduction of uric acid levels between the treatment and control groups. TLS: RR (95% CI) = 0.77 (0.44–1.33)	14
Alshehri et al., 2021^39^	Medline, Google Scholar, Scopus, Embase, and Cochrane from inception till April 2021	Not provided	K = 20 Control trial (3) RCT (3) Cohort (3) Retrospective (11) RoB: Newcastle–Ottawa scale (NOS)	Rasburicase	Development of TLS (6) White blood cell count (1) Lactate dehydrogenase (2) Uric acid level (13) Creatinine (7) AKI (1) Adverse events (6)	Odds ratio for the development of TLS 4.06 (3.11–5.28). A significant main effect of rasburicase for the development of TLS, uric acid level (MD = −19.96 (−22.43 to −17.49), and creatinine level (*p* < 0.01)	8

AKI: Acute kidney injury; AMSTAR 2: A measurement tool to assess systematic reviews 2; CCT: Controlled clinical trail; CI: Confidence interval; CTLS: Clinical tumor lysis syndrome; EMBASE: Excerpta Medica Database; ER: Effect ratio; ICU: Intensive care unit; LTLS: Laboratory tumor lysis syndrome; MD: Mean difference; MEDLINE: Medical Literature Analysis and Retrieval System Online; NOS: Newcastle–Ottawa Scale; OR: Odds ratio; PUA: Plasma uric acid; RCT: Randomized controlled trial; RoB: Risk of bias; RR: Risk ratio; RRT: Renal replacement therapy; SDR: Single-dose rasburicase; TLS: Tumor lysis syndrome.

### Risk of bias assessment

The risk of bias of the included studies was assessed using the AMSTAR 2 tool, followed by qualitative and quantitative syntheses.

Except for four studies, most of the reviews defined their review questions according to the PICO format, defined the method before conducting the review, explained the study design for the review, selected studies, extracted data based on the agreement of two reviewers, and conducted risk of bias assessments.^33^^,^^34^^,^^36^^,^^37^ The search strategy was not reported appropriately in one review.^37^ Only one review provided a list of excluded studies, the impact of RoB on quantitative and qualitative synthesis, and publication bias.^38^ None of the included reviews reported the funding sources for the included studies. Three reviews discussed study heterogeneity,^31^^,^^33^^,^^38^^,^ and the source of conflict was declared in five of the included reviews.^32^^,^^33^^,^^35^^,^^37^^,^^39^ Table 2 illustrates the quality appraisal using AMSTAR 2.

**Table 2:** AMSTAR 2 quality appraisal for all studies.

Author, year	PICO	Methods before conducting the study	Explanation of the study design	Search strategy	Study selection	Data extraction	Exclusion	Adequate detail of studies	RoB	Source of funding	Appropriate method for statistical combination	Impact of RoB on the Meta-analysis	RoB in discussion	Heterogeneity in studies	Publication bias	Source of conflict	Score
Lopez-Olivo et al., 2013^31^	Yes	Partially yes	Yes	Yes	Yes	Yes	No	Yes	Yes	No	Yes	No	No	Yes	No	No	9
Yu et al., 2017^32^	Yes	Yes	Yes	Partially yes	Yes	Yes	No	Yes	Yes	No	Yes	No	No	No	No	Yes	9
Dinnel et al., 2015^33^	No	No	No	Partially yes	No	No	No	Yes	No	No	N/A	N/A	Yes	Yes	N/A	Yes	4
Feng et al., 2013^34^	No	No	Yes	Yes	No	No	No	Yes	No	No	Partially yes	N/A	N/A	No	N/A	No	3
Masamayor et al., 2022^35^	Yes	Yes	Yes	Yes	Yes	Yes	No	Yes	Yes	No	Yes	No	No	No	No	Yes	10
Bose et al., 2011^36^	No	No	No	Yes	No	No	No	Yes	No	No	N/A	N/A	N/A	No	N/A	No	2
Alakel et al., 2017^37^	No	No	No	No	No	No	No	No	No	No	N/A	N/A	N/A	No	N/A	Yes	1
Cheuk et al., 2017^38^	Yes	Yes	Yes	Yes	Yes	Yes	Yes	Yes	Yes	No	Yes	Yes	Yes	Yes	Yes	No	14
Alshehri et al., 2021^39^	Yes	Yes	Yes	Partially yes	Yes	Partially yes	No	Yes	Yes	No	Yes	No	No	No	No	Yes	8

N/A: Not applicable; PICO: Population, intervention, comparator, outcome; RoB: Risk of bias.

To evaluate the quality of the evidence, the AMSTAR 2 scores were evaluated as confidence categories, including high, moderate, low, and critically low [Table 3].

**Table 3:** Study grades according to AMSTAR 2.

Author (year)	Grade (Confidence category)
Lopez-Olivo et al., 2013^31^	Moderate
Yu et al., 2017^32^	Moderate
Dinnel et al., 2015^33^	Critically low
Feng et al., 2013^34^	Critically low
Masamayor et al., 2022^35^	Moderate
Bose et al., 2011^36^	Critically low
Alakel et al., 2017^37^	Critically low
Cheuk et al., 2017^38^	High
Alshehri et al., 2021^39^	Moderate

The search strategy for including reviews was conducted from inception to December 2021. The databases were PubMed, EMBASE, MEDLINE, Web of Science, ClinicalTrails.gov, Scopus, Google Scholar, CINAHL (Cumulative Index to Nursing and Allied Health Literature), and the Cochrane Central Register of Controlled Trials (CENTRAL). Five of the nine included reviews assessed the risk of bias of the included studies. Of these five reviews, four used the Cochrane Collaboration’s Risk of Bias Assessment tool, while one used the Newcastle–Ottawa scale (NOS).

The total sample size of the included reviews was 3331 for both the intervention and control groups. Four reviews did not mention the total sample size of the included studies.^33^^,^^36^^,^^37^^,^^39^ The sample size for one of the studies (*n* = 807) by Bose et al.^36^ was calculated manually from the included studies, resulting in a combined sample size of the umbrella review of 4138. Only two reviews mentioned the population (children or adults),^33^^,^^39^ while another review mentioned neither population nor sample size, as no meta-analysis was conducted.^39^ Three reviews included both children and adults as samples,^32^^,^^33^^,^^39^ whereas the other reviews were conducted on adult patients.

This review included a total of 213 studies. Among these included studies were observational studies (27), retrospective studies (77), RCTs (18), clinical controlled trials (5), prospective studies (30), reviews (50), case series (3), and cohort studies (3).

Regarding the intervention, four of the nine included reviews explored the efficacy of a single dose of rasburicase for TLS outcomes,^33^^,^^36^^,^^37^^,^^39^ while two reviews compared the efficacy of a single dose *vs.* a weight-based dose of rasburicase in terms of TLS outcomes.^32^^,^^34^ The single doses of rasburicase were 1.5, 3, 4.5, 6, and 7.5 mg per day, while the weight-based doses were 0.05 and 0.15 mg/kg. Three reviews compared the efficacy of rasburicase with allopurinol^31^^,^^34^^,^^38^ in reducing TLS outcomes, and one review explored the efficacy of urate oxidase, including rasburicase, with intravenous hydration using crystalloid solutions and sodium bicarbonate on TLS outcomes.^35^

This review examined a wide variety of TLS outcomes. The outcomes included TLS development (26 studies), white blood cell counts (one study), lactate dehydrogenase level (two studies), disease prognosis (16 studies), adverse events (39 studies), AKI (25 studies), mortality (four studies), mortality due to TLS (four studies), serum uric acid level (91 studies), creatinine levels (14 studies), the incidence of RRT (24 studies), admission or length of stay in the intensive care unit (ICU) (two studies), tolerability (two studies), and dose reduction (12 studies).

### TLS development

The results of RCTs on the development of TLS are contradictory. Two reviews reported that rasburicase, as compared to allopurinol, has a significant effect on the development of TLS among patients (risk ratio [RR] = 0.74, 95% CI = 0.17–3.22;^35^ OR = 4.06, 95% CI = 3.11–5.28),^39^ while two reviews reported no difference in the incidence of TLS (RR = 0.74, 95% CI = 0.17–3.20;^31^ RR = 0.77, 95% CI = 0.44–1.33).^38^ The meta-analysis exploring the difference in the efficacy between rasburicase and allopurinol excluded a study by Feng et al.^34^ because of the low certainty of the evidence. The results of the meta-analysis of the meta-analyses indicated no significant difference in efficacy between rasburicase and allopurinol in TLS development (RR = 0.98, 95% CI = 0.74–1.31, *p* > 0.05) [Figure 2].

According to observational studies, 7.40% (95% CI = 1.70–16.70%) of patients experienced TLS even after rasburicase administration;^31^ however, a 7.5 mg/day dose of rasburicase was the most effective in reducing the incidence of TLS among patients (98.6%, 95% CI = 0.90–1.01).^32^ However, other studies reported no difference in the efficacy of single and daily doses of rasburicase against TLS development;^34^ nevertheless, the quality of evidence was low. The combined effect size (RR) for the observational study was 0.07 (95% CI = 0.02–0.25).^31^ The meta-analysis of the meta-analyses also established the efficacy of rasburicase against TLS development; however, the effect was not significant (RR = 0.76, 95% CI = 0.20–2.87, *p* > 0.05) [Figure 3]. The efficacy of single-dose rasburicase over that of allopurinol in reducing the risk for laboratory and clinical TLS was established in retrospective and prospective studies (OR = 3.83, 95% CI = 2.17–6.76),^33^^,^^34^^,^^36^ but the quality of evidence was not robust. The response rate for rasburicase reported in retrospective and prospective studies (effect ratio [ER]) was 0.87 (95% CI = 0.82–0.93).^32^

### Uric acid levels

**Figure 4:**
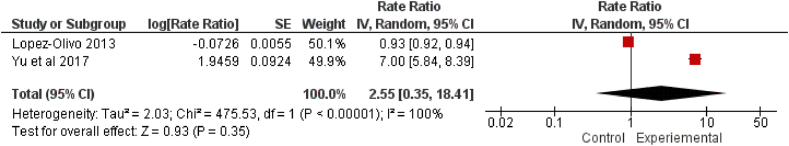
Efficacy of rasburicase in reducing serum uric acid level. CI: Confidence interval. df: Degrees of freedom; IV: Inverse variance; SE: Standard error.

RCTs and clinical controlled trials indicated that rasburicase had a significant main effect on reducing and maintaining serum uric acid levels compared to the effects of allopurinol (mean difference [MD] = −569 mg/dL, 95% CI = [−796.38,−341.62] mg/dL^35^; MD = −201.00 mg/dL, 95% CI = [−258.05, −143.95] mg/dL^38^; MD = −19.96 mg/dL, 95% CI = [–22.43,–17.49] mg/dL^39^). Observational studies reported that 93.4% of patients achieved normal serum uric acid levels,^31^ and 6 mg and 0.15 mg/kg doses of rasburicase were most effective in serum uric acid reduction, with a mean reduction of 8.45 mg/dL (95% CI = 7.51–9.38 mg/dL) and 10 mg/dL (95% CI = 8.58–11.42 mg/dL), respectively.^32^ The combined estimated effects of the observational studies were RR = 0.93 (95% CI = 0.92–0.95)^31^ and ER = 7.0 (95% CI = 5.84–8.17).^32^ Meta-analyses also reported that while rasburicase reduced serum uric acid levels compared to those of the control group, the change was not significant (RR = 2.55, 95% CI = 0.35–18.41, *p* = 0.35) [Figure 4]. The results of prospective and retrospective studies indicated that rasburicase significantly controlled serum uric acid levels compared to that observed with allopurinol;^33^^,^^36^ the quality of the evidence was significantly low. Moreover, a daily dose of rasburicase resulted in a greater reduction of uric acid levels than that with a single dose of rasburicase.^36^

### Adverse events

RCTs reported no difference in adverse events between the rasburicase and allopurinol groups (RR = 0.54, 95% CI = 0.12–2.48).^38^ Observational studies reported that 2.60% (95% CI = 1.70–3.80%) of patients experienced adverse events, with an RR of 0.03 (95% CI = 0.02–0.04).^31^ The most commonly reported adverse events were related to the blood, lymphatic, and gastrointestinal systems.^35^ The quality of all evidence was high.

### Mortality

No robust evidence suggests that rasburicase reduced mortality among patients with TLS.^33^^,^^38^ Observational studies reported that 2.60% (95% CI = 0.90–5.00%) of patients died after rasburicase administration, with a combined effect estimate, i.e., RR = 0.03 (95% CI = 0.01–0.05).^31^

### Renal replacement treatment

The dose regimen of 4.5 mg resulted in significantly lower RRT incidence than did other dose regimens (ER = 0.22, 95% CI = 0.15–0.29) with the combined effect of all dose regimens, i.e., ER = 0.65 (95% CI = 0.03–0.10).^32^

### Economic outcomes

The results of the included reviews established the cost-effectiveness of rasburicase, as it reduced the administration frequency and duration of ICU stay.^33^ Moreover, they established that single-dose rasburicase is the most cost-effective drug for treating TLS.^33^^,^^34^ However, whether rasburicase is economically superior to allopurinol remains unknown.

## Discussion

This umbrella review synthesized the literature on the efficacy of rasburicase against TLS development and in reducing uric acid levels. The results indicated that although rasburicase reduced the risk of TLS development, rasburicase did not differ significantly from the control (including allopurinol) in reducing the risk of TLS development. The included reviews also reported that rasburicase significantly reduced serum uric acid levels; however, the reduction in serum uric acid did not differ significantly between rasburicase and allopurinol. Similarly, rasburicase did not reduce the mortality rate, adverse events, or RRT incidence.

Rasburicase reduces serum uric acid levels but does not significantly affect TLS development.^40^ Limited literature is available on the efficacy of rasburicase in preventing TLS among low-risk patients; however, the absence of an advantage of rasburicase in reducing renal failure among high-risk patients raises skepticism about its potential efficacy among low-risk patients. Some of the results align with previous literature by showing the 7.5 mg/day dose to be the most efficacious in reducing TLS outcomes,^27^ but others contradict the literature by indicating that rasburicase does not reduce the number of inpatient days.^41^

Niforatos et al.^42^ analyzed a previously conducted head-to-head RCT to identify the differences in serum uric acid reduction by rasburicase, rasburicase plus allopurinol, and allopurinol alone. The results indicated significant reductions in serum uric acid levels in all three arms but no change in other outcomes, including TLS development, increased creatinine levels, AKI, and renal failure. A similar finding was reported by Martens et al.^43^ in their retrospective analysis, which showed no difference in TLS development, AKI, RRT, or creatinine levels between patients receiving rasburicase and allopurinol. Although rasburicase has been associated with greater serum uric acid reduction than was allopurinol, according to meaningful RCTs^38^ described earlier, this efficacy of rasburicase does not translate into clinically meaningful risk reduction. The literature further suggests that rasburicase does not prevent renal injury or mortalities.^44^

Rasburicase is an expensive medicine, costing $37,500. Most studies on the efficacy of rasburicase usually include high-risk patients in their sample, resulting in an overestimation of the cost-effectiveness of rasburicase among patients who are at low to intermediate risk of developing TLS.^45^^,^^46^ Its highly established efficacy makes physicians more prone to inappropriately prescribe rasburicase to patients at low to intermediate risks of developing TLS. The inappropriate use of rasburicase ranges from 32% to 70% and includes prescriptions in cases of hyperuricemia without evidence of high-risk TLS, no trials of allopurinol, and no renal failure.^45^^,^^46^ Additionally, several side effects are associated with using rasburicase, with 1% of patients experiencing rasburicase-induced anaphylaxis and 20–50% experiencing viral syndrome-like and gastrointestinal symptoms.^47^ The side effects of rasburicase also include pruritus, nausea, gout, maculopapular rash, and renal failure, which occur in 1–10% of patients.

Our review highlights the need for future research focusing on the cost-effectiveness of rasburicase compared with that of allopurinol, which is less expensive and has fewer side effects. This information can help inform clinical decision-making by considering the relative costs and benefits of each medication and can potentially lead to more efficient and cost-effective use of healthcare resources. Our recommendation is to stratify patients based on risk in future large-scale trials, which can also help ensure that the findings are generalizable to a broader population of patients with malignancies.

This review may be subject to publication bias, which may skew the results. The meta-analyses included in this umbrella review may have used different study designs, populations, and outcome measures, which may have limited the ability to draw meaningful conclusions across studies. Moreover, the included meta-analyses may have used different methods of data extraction, analysis, and synthesis, which may have affected the quality and validity of the results. Additionally, the quality and reporting of individual studies included in the meta-analyses may have been inconsistent, which could have affected the accuracy of the findings. Some of the included meta-analyses had limited sample sizes, which may have affected the statistical power and precision of the results. The review was limited to studies published in English, and relevant studies from non-English language sources were excluded. Finally, the included meta-analyses may have been subject to funding bias, where the funding source could influence the study design, conduct, or reporting.

## Conclusion

The analysis of the evidence and associated literature demonstrated that rasburicase is effective in reducing TLS development and serum uric acid levels in children and adults with malignancies; however, its effect does not differ significantly from that of allopurinol, which has greater cost-effectiveness and fewer side effects. Future trials should focus more on the cost-effectiveness of rasburicase compared to that of allopurinol and include high-, intermediate-, and low-risk patients.

## Funding

None.

## Authors contribution

Kamran Mahfooz: Conceptualization, Writing original draft, Data extraction, Data Analysis, Project Administration; Haris Sohail: Conceptualization, Writing original draft, Data extraction; Ani Gvajaia: Conceptualization, Writing original draft, Data extraction; Uroosa Arif: Conceptualization, Writing original draft, Data extraction; Daisy Grewal: Conceptualization, Writing original draft, Data extraction; Monica Reddy Muppidi: Conceptualization, Writing original draft, Data extraction; Vanya Vohra: Conceptualization, Writing original draft, Data extraction; Aamir Tarique: Conceptualization, Writing original draft, Data extraction; Advait Vasavada: Conceptualization, Writing original draft.

## Ethics statement

None.

## Data availability statement

The datasets used in the current study are available from the corresponding author on reasonable request.

## Conflict of interest

None.
